# Potential of *Oscheius tipulae* nematodes as biological control agents against *Ceratitis capitata*

**DOI:** 10.1371/journal.pone.0269106

**Published:** 2022-06-07

**Authors:** Ameni Loulou, Meriem M’saad Guerfali, Arthur Muller, Aashaq Hussain Bhat, Joaquín Abolafia, Ricardo A. R. Machado, Sadreddine Kallel

**Affiliations:** 1 Laboratory of Bio-aggressor and Integrated Protection in Agriculture, Department of Plant health and Environment, National Agronomic Institute of Tunisia, University of Carthage, Tunis, Tunisia; 2 Laboratory of Biotechnology and Nuclear Technologies, National Center of Nuclear Sciences and Technologies, Technopole Sidi Thabet, Ariana, Tunisia; 3 Faculty of Sciences, Experimental Biology Research Group, Institute of Biology, University of Neuchâtel, Neuchâtel, Switzerland; 4 Department of Zoology, Government Degree College, Kathua, Jammu, Jammu and Kashmir, India; 5 Departamento de Biología Animal, Biología Vegetal y Ecología, Universidad de Jaén, Jaén, Spain; University of Carthage, TUNISIA

## Abstract

A survey to collect soil nematodes with potential to control *Ceratitis capitata* flies was carried out in different locations in Tunisia. Several nematode isolates were recovered, laboratory colonies were established, and their taxonomic identities were determined based on molecular methods. Among all the recovered nematode isolates, two of them, *Oscheius tipulae* TC2 and OC2, were evaluated for their capacity to control *C*. *capitata* flies and for their ability to kill and reproduce on *Galleria mellonella* larvae. Our results show a great potential of these two isolates as biocontrol agents as they kill *C*. *capitata* eggs and pupae and interfere with the metamorphosis of *C*. *capitata* larvae. More specifically, TC2 and OC2 nematodes killed 39 and 31% of *C*. *capitata* eggs, respectively, impaired the metamorphosis of up to 77% and up to 67% of *C*. *capitata* larvae, respectively, and killed up to 66% and up to 58% of *C*. *capitata* pupae, respectively. The efficacy of TC2 and OC2 nematodes was particularly high on *C*. *capitata* pupae, and significant insect mortalities were observed even at concentrations of 1 and 5 nematodes/pupae, respectively. We also found that TC2 and OC2 nematodes efficiently kill and reproduce in *G*. *mellonella* larvae, suggesting that these insects could be used for mass-multiplication of these nematodes. These results reveal the potential of *O*. *tipulae* to complement integrated pest management programs against *C*. *capitata* flies.

## Introduction

The citrus agro-industry is one of the most important sectors for the economy of Tunisia. Citrus production is unfortunately hampered by the occurrence of different diseases and pests that considerably impair tree growth and crop yields. *Ceratitis capitata* (Wiedemann) (Diptera: Tephritidae), the Mediterranean fruit fly or medfly, is one of the most limiting insect pests for citrus production [1]. Several cultivated citrus species in Tunisia such as *Clementine*, *Mandarin*, *Navel*, *Maltaise*, *Valencia Late* and *Double Fine* oranges are attacked by *C*. *capitata* although to different degrees of intensities [2,3]. This insect species is considered as one of the most devastating pests in the world, and causes damage not only to citrus plants, but also to many other cultivated and not-cultivated plant species [2]. The control of medfly in Tunisia is based on the spraying of broad-spectrum chemical pesticides, such as the highly toxic organophosphates [3]. The use of these chemicals is problematic as they cause environmental pollution, affect the health of animals and humans, and does not provide long-term solutions for agricultural problems, as insect pests have the capacity to evolve resistance to these chemicals [4,5]. The use of biocontrol agents is proposed as a more environmentally friendly control strategy against insect pests, as it can provide long-term solutions to reduce the negative socio-economic impact of Mediterranean fruit flies [6,7].

From the great variety of biocontrol agents, entomopathogenic nematodes (EPNs) are highly promising due to their capacity to efficiently control different insect pests [8–10]. The most important groups of EPNs for use as biocontrol agents belong to two nematode genera: *Steinernema* (Travassos) (Nematoda: Rhabditida) and *Heterorhabditis* (Poinar) (Nematoda: Rhabditida), which are associated with bacteria of the genus *Xenorhabdus* and *Photorhabdus*, respectively [11–17]. Several species of these nematodes are currently being mass-produced and used to control a number of soil-dwelling insect pests [18–20]. Apart from these two predominant groups, several other nematode species have also shown the potential to kill insects, as it is the case with nematodes of the genus *Oscheius* (Andrássy) (Nematoda: Rhabditidae) [21–30].

*Oscheius* nematodes are soil-dwelling nematodes of the Rhabditidae family. Several of the species of this genus are considered scavengers, or bacteriophagous free-living nematodes, while several others have been shown to kill insects. Currently, there are about 30 recognized species in the genus *Oscheius*, and more than 13 have the ability to kill insects [21,22]. For instance, *Oscheius onirici* kills the larvae of *Galleria mellonella* (Linnaeus) (Lepidoptera: Pyralidae), *Tenebrio molitor* (Linnaeus) (Coleoptera: Tenebrionidae) and *Drosophila suzukii* (Matsumura) (Diptera: Drosophilidae); *Oscheius carolinensis* has potential as a biological control agent against *Pieris rapae* (Linnaeus) (Lepidoptera: Papilionoidae) and *T*. *molitor*; *Oscheius gingeri* was efficient against *G*. *mellonella* and *Conogethes punctiferalis* (Guenée) (Lepidoptera: Crambidae) larvae; and *Oscheius tipulae* and *O*. *rugaoensis* also kill *G*. *mellonella* larvae [14,23–30]. This trait seems not to be limited to the above-mentioned species, and the number of studies reporting the biocontrol potential of several species of this genus is steadily growing [31]. However, there is some intraspecific variation in their insect-killing abilities. For instance, several *O*. *onirici* isolates were shown to kill different insect species, while a Swiss isolate was not harmful to *G*. *mellonella* [32,33]. There are some species, such as *O*. *saproxylicus*, and *O*. *tereticorpus*, whose ability to kill insects have not been tested, however, suggesting that this trait could be much more spread in the genus than it is currently thought [22,27,32–40]. Clearly, several species of this nematode genus have great potential to complement the repertory of commercially available *Steinernema* and *Heterorhabditis* nematodes to control agricultural pests.

Given the promising potential of *Oscheius* nematodes as biocontrol agents and aiming at increasing their availability to be used to control *C*. *capitata* flies, we collected soil-dwelling nematodes at several locations in Tunisia using *C*. *capitata* pupae as baits, characterized them using molecular tools to determine their taxonomic identities, and selected *Oscheius* nematodes to specifically evaluate their abilities to kill *C*. *capitata* at different developmental stages and temperatures. As *C*. *capitata* pupates in the soil, it is one of the most suitable stages for biocontrol using soil-born nematodes such as *O*. *tipulae*. The objective of our study was to show the great biocontrol potential of two *O*. *tipulae* isolates against *C*. *capitata* flies. Hence, our study encourages to continue studying the biology of *O*. *tipulae* nematodes and their interaction with *C*. *capitata* and other insect pests to determine their actual biocontrol potential and to explore the possibility of incorporating them in biocontrol programs against agricultural pests.

## Materials and methods

### Soil sampling and nematode isolation

Soil samples were collected, during the winter season of 2019 and 2020, at five different locations in Tunisia: Morneg, Takilsa, Kobba, Sidi-Saad, and Ouzra. Soil samples were taken from soils of citrus crops (*Citrus sinensis* var. Maltaise) infested by *C*. *capitata*. Twenty soil samples per location were collected at a depth of 0-30cm using a hand shovel. Samples were placed in plastic bags and stored at 7°C for further analyses. Soil nematodes were recovered from the soil samples using *C*. *capitata* pupae or greater wax moth larvae as baits according to the procedures described by Bedding and Akhurst [41]. *Ceratitis capitata* pupae were included as baits to obtain nematodes closely associated to this pest. For each sample, 300g of soil were placed in 500ml plastic containers. Then, ten *C*. *capitata* pupae, reared in artificial diets as described below, were added to the plastic containers. Soil was moistened using a water sprayer. Plastic containers were incubated at 22°C in the dark for 5 days. After this period, all dead insects were collected, rinsed three times with distilled water and incubated for 24 hours at ambient temperature. Then, five *G*. *mellonella* larvae were added to the same soil container. Plastic containers were incubated at 22°C in the dark for 5 days. All dead insects were collected, rinsed three times with distilled water, incubated for 24 hours at ambient temperature, transferred to White traps and incubated at 22°C for 8 days in darkness [42]. The presence of nematodes was checked every 2 days. Emerging nematodes were collected and used to infest *C*. *capitata* pupae and *G*. *mellonella* larvae, or were cultured in egg yolk media (24g egg yolk, 12g agar in 500ml distilled water). In all cases, cultures were maintained in darkness at 25°C. Progenies were collected and cultured in fresh media/insects. After several cycles, the nematode cultures were inspected under a light microscope (Olympus^®^, model SZX-ILLK200, Japan) to determine potential mixture of different nematode species.

### Insect rearing

Mediterranean fruit flies were obtained from a stock colony of the Vienna-8 genetic sexing strain maintained at the laboratory of sterile insects at the National Centre of Nuclear Sciences and Technologies of Tunisia (CNSTN) [43]. Adult flies were kept in cages with two sides covered with a mesh for oviposition. Adults were fed artificial diets composed of yeast hydrolyzate and water (3:1 ratio). Eggs were collected daily from water containers covered with a mesh fabric. Trays containing sawdust were provided for pupation [44]. *Galleria mellonella* larvae were collected from naturally infested beehives.

### Nematode identification

Nematode genomic DNA was extracted from about five thousand nematodes at all developmental stages using the genomic DNA isolation kit from NORGEN BioTEK (Cat. 24700) following the manufacturer’s instructions. Genomic DNA was used to amplify different regions of the rRNA genes by PCR. Briefly, ITS regions (ITS1, 5.8S, ITS2) were amplified using primers 18S: 5’-TTGATTACGTCCCTGCCCTTT-3’ (forward), and 26S: 5’-TTTCACTCGCCGTTACTAAGG-3’ (reverse) [45]. The fragment containing the D2/D3 regions of the 28S rRNA gene was amplified using primers D2F: 5’-CCTTAGTAACGGCGAGTGAAA-3’ (forward) and 536: 5’-CAGCTATCCTGAGGAAAC-3’(reverse). The 18S rRNA gene was amplified using primers NEM18SF: 5’-CGCGAATRGCTCATTACAACAGC-3’ (forward) and NEM18SR: 5’-GGGCGGTATCTGATCGCC-3’ (reverse) [46]. PCR cycling conditions used were: an initial denaturation step at 98°C for 10min, annealing at 58°C for 30s and extension at 72°C for 90s. PCR products were separated by electrophoresis (45 min, 100 volts) in a 1% TAE (Tris–acetic acid–EDTA) buffered agarose gel stained with GelRed nucleic acid gel stain (Biotium). PCR products were sent to Microsynth AG (Balgach, Switzerland) for Sanger sequencing. Sequences were manually curated and trimmed. All sequences were deposited in the National Center for Biotechnology Information (NCBI) databank. Accession numbers are given in the phylogenetic trees and summarized in S1 Table.

### Phylogenetic relationships reconstruction

The evolutionary histories based on the different rRNA gene sequences were inferred by using the Maximum Likelihood method based on the General Time Reversible model (18S and D2D3) or on the Tamura 3-parameter model (ITS) [47,48]. Best-fit substitution model analyses were carried out prior to inferring evolutionary histories [48,49]. In all cases, the trees with the highest log likelihood are shown. The percentage of trees in which the associated taxa clustered together is shown next to the branches. Initial tree(s) for the heuristic search were obtained automatically by applying Neighbor-Join and BioNJ algorithms to a matrix of pairwise distances estimated using the Maximum Composite Likelihood (MCL) approach, and then selecting the topology with superior log likelihood value. A discrete Gamma distribution was used to model evolutionary rate differences among sites (5 categories). The trees are drawn to scale, with branch lengths measured in the number of substitutions per site. Evolutionary analyses were conducted in MEGA7 [49]. Graphical representation and editing of the phylogenetic trees were performed with the Interactive Tree of Life (version 3.5.1) [50,51].

### Biocontrol potential of *O*. *tipulae* against different developmental stages of *C*. *capitata*

To evaluate the potential of *O*. *tipulae* nematodes to control *C*. *capitata*, we evaluated metamorphosis and mortality of *C*. *capitata* eggs, larvae, and pupae exposed to different concentrations of two *O*. *tipulae* isolates, TC2 and OC2, as described below.

#### Biocontrol potential of *O*. *tipulae* nematodes against *C*. *capitata* eggs

The biocontrol potential of *O*. *tipulae* TC2 and OC2 nematodes on *C*. *capitata* eggs was evaluated based on the induced mortality of these latter. Fifty eggs were placed in sterile Petri plates (60 mm diameter) layered with two filter paper sheets. Then, 5000 *O*. *tipulae* TC2 or 5000 OC2 nematodes suspended in 2ml of sterile water were added to each Petri plate. Controls were treated with water only. Then, all Petri plates were sealed with Parafilm. Six Petri plates for each treatment were evaluated (n = 6). Egg mortalities, measured as the number of eggs that did not hatch, were recorded three days post-treatment. The bioassay was maintained at 22°C.

#### Biocontrol potential of *O*. *tipulae* nematodes against *C*. *capitata* larvae

The biocontrol potential of *O*. *tipulae* TC2 and OC2 nematodes on *C*. *capitata* larvae wa evaluated based on successful metamorphic transition of *C*. *capitata* larvae. Twenty third instar larvae were placed in sterile Petri plates (60 mm diameter) layered with two filter paper sheets. Then, either 50, 125, 250 or 500 nematodes/larva suspended in 1 ml distilled water were added to each Petri plate. Controls were treated with pure water only. Then, all Petri plates were sealed with Parafilm. Five Petri plates for each treatment were evaluated (n = 5). Due to the rapid transition from the larval to the pupal stage, the number of healthy and unhealthy pupae was recorded three days post-treatment. The bioassay was maintained at 25°C.

#### Biocontrol potential of *O*. *tipulae* nematodes against *C*. *capitata* pupae

The biocontrol potential of *O*. *tipulae* TC2 and OC2 nematodes on *C*. *capitata* pupae was evaluated based on the induced mortality of these latter. Six nematode doses were used: 1, 5, 10, 50, 100 and 500 nematodes/pupa suspended in 1 ml of water. Controls were treated with pure water only. For each treatment, 5 Petri plates (60 mm diameter) layered with two filter paper sheets and with 20 pupae each were assayed. All Petri plates were sealed with Parafilm. The bioassays were maintained at 25°C.

#### Impact of temperature on the biocontrol potential of *O*. *tipulae*

To evaluate the impact of temperature on the biocontrol potential of *O*. *tipulae* TC2 and OC2 nematodes, the mortality of *C*. *capitata* pupae exposed to different nematode concentrations was evaluated at different temperatures. Four different nematode concentrations were used: 50, 125, 250 and 500 nematodes/pupa suspended in 1 ml of distilled water. Controls were treated with pure water only. Five Petri plates (60 mm diameter) layered with two filter paper sheets and with twenty 3- to 5-day-old pupae each were assayed. All Petri plates were sealed with Parafilm. The bioassays were maintained at 20, 25, and 30°C.

### Reproduction potential of *O*. *tipulae* nematodes on *G*. *mellonella* larvae

To evaluate the potential of *O*. *tipulae* TC2 and OC2 nematodes to kill and reproduce on *G*. *mellonella*, the mortality of *G*. *mellonella* larvae and the number of emerging nematodes from *G*. *mellonella* larvae exposed to these nematodes was evaluated in three independent experiments. In the first experiment, Petri plates (110mm diameter) were layered with two filter paper sheets, then ten first-instar *G*. *mellonella* larvae were placed in each plate and treated with either *O*. *tipulae* TC2 or OC2 nematodes at a concentration of 500 nematodes/larva suspended in 1 ml of water. Four Petri plates per nematode strain were used. In the second experiment, Petri plates (60 mm diameter) were each layered with two filter paper sheets, then one first-instar *G*. *mellonella* larva was placed in each Petri plate and treated with either *O*. *tipulae* TC2 or OC2 nematodes at a concentration of 500 nematodes/larva suspended in 1ml of water. Ten Petri plates per nematode strain were used. In the third experiment, Petri plates (60 mm diameter) were layered with two filter paper sheets, then ten first-instar *G*. *mellonella* larvae were placed in each Petri plate and treated with either *O*. *tipulae* TC2 or OC2 nematodes at a concentration of either 250 or 500 nematodes/larva suspended in 1 ml of water. Three Petri plates per nematode strain and concentration were used. In all experiments, controls were treated with pure water only. Insect mortality was recorded every 24h for five days. To evaluate the reproductive potential of nematodes, all dead larvae from the third experiment were rinsed with water and individually placed in White traps [52]. Emerging nematodes were counted ten days after. All bioassays were maintained at 25°C in darkness.

### Statistical analysis

Differences in egg mortalities were assessed by one-way ANOVA with nematode isolate as a factor. Differences in the number of deformed pupae and pupal mortalities were assessed by two-way ANOVA with nematode isolate and nematode concentration as factors. The effect of temperature on nematode biocontrol potential against *C*. *capitata* pupae was assessed by three-way ANOVA with temperature, nematode isolate and nematode concentration as factors. *Galleria mellonella* mortalities were analyzed by two-way repeated measures ANOVA with time and nematode isolate as factors. Nematode reproduction was evaluated by two-way ANOVA with nematode isolate and nematode concentration as factors. Normality and equality of variance were verified using Shapiro–Wilk and Levene’s tests, respectively. Holm–Sidak post hoc tests were used for multiple comparisons. All statistical analyses were conducted using Sigma Plot 14.5 (Systat Software Inc., San Jose, CA, USA).

## Results

### Nematode isolation and identification

**Fig 1:**
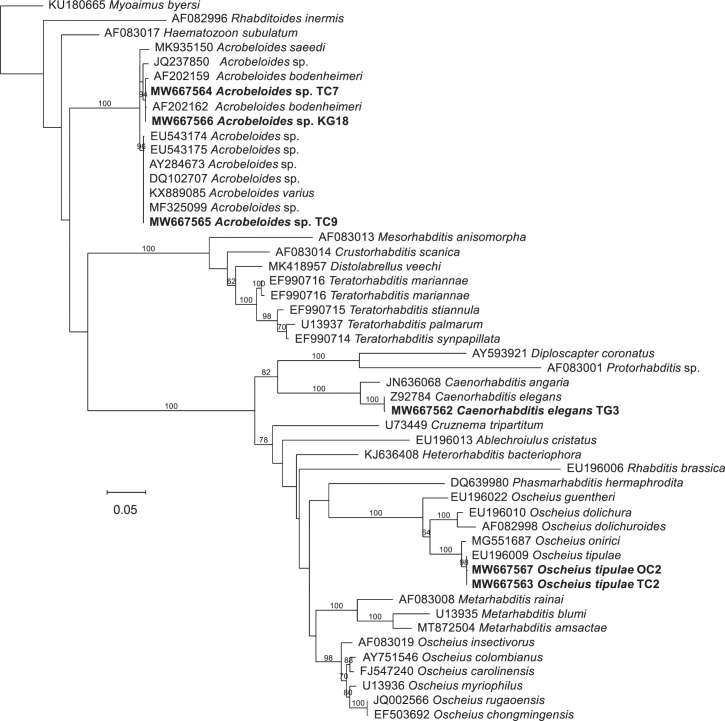
Phylogenetic tree based on ribosomal DNA sequences of the nematodes isolated in this study and several related species. Phylogenetic relationships based on 18S rRNA gene sequences were inferred by using the Maximum Likelihood method based on the General Time Reversible model. The tree with the highest log likelihood (-9560.62) is shown. The percentage of trees in which the associated taxa clustered together is shown next to the branches. A discrete Gamma distribution was used to model evolutionary rate differences among sites (5 categories (+*G*, parameter = 0.8832)). The tree is drawn to scale, with branch lengths measured in the number of substitutions per site. NCBI accession numbers of the sequences used for the analyses are shown.

Laboratory colonies were successfully established from six out of the 100 soil samples collected (Table 1). Based on the analysis of their 28S rRNA gene sequences, the nematode isolates recovered were identified as *O*. *tipulae* (TC2, OC2), *Caenorhabditis elegans* (TG3), and *Acrobeloides* spp. (TC7, KG18, and TC9) (Fig 1, S1 and S2). *Acrobeloides* sp. TC7 and KG18 could tentatively be identified as *A*. *bodenheimeri* based on the 18S rRNA gene sequences (Fig 1). However, as the availability of sequences for *Acrobeloides* nematodes are very limited, and most of the molecular data available are not linked to morphological data, the species identity of *Acrobeloides* sp. nematodes awaits confirmation based on morphological data. The two populations of *O*. *tipulae* were isolated from *C*. *capitata* pupae in the regions of Takilsa and Ouzra. *Caenorhabditis elegans* was isolated from *G*. *mellonella* larvae and was found in only one soil sample from Takilsa. *Acrobeloides* nematodes were obtained from the region of Takilsa and Kobba, and isolated from *C*. *capitata* pupae and from *G*. *mellonella* larvae. No nematodes were recovered from the soil samples collected in the regions of Morneg and Sidi-Saad (Table 1).

**Table 1:** Geographical location of nematode sampling sites. Soil samples were collected at five different locations in Tunisia: Takilsa, Ouzra, Kobba, Sidi-Saad, and Morneg. Place of sample collection, GPS coordinates, number of samples collected, nematode species isolated, strain name designation and insect host of the different nematode strains isolated.

Place of sample collection	GPS coordinates	Number of soil samples collected	Nematode species isolated	Designated strain name	Insect host
Takilsa	36°47’30.5"N 10°37’32.1"E	20	*O*. *tipulae*	TC2	*C*. *capitata* pupae
			*Acrobeloides* sp.	TC7	*C*. *capitata* pupae
			*Acrobeloides* sp.	TC9	*C*. *capitata* pupae
			*C*. *elegans*	TG3	*G*. *mellonella* larvae
Ouzra	36°38’47.0"N 10°14’41.3"E	20	*O*. *tipulae*	OC2	*C*. *capitata* pupae
Kobba	36°37’17.6"N 10°32’45.0"E	20	*Acrobeloides* sp.	KG18	*G*. *mellonella* larvae
Sidi-Saad	36°40’5.9"N 10°16’17.6"E	20	none	-	-
Morneg	36°38’28.0"N 10°13’2.8"E** **	20	none	-	-

### Biocontrol potential of *O*. *tipulae* nematodes against *C*. *capitata*

**Fig 2:**
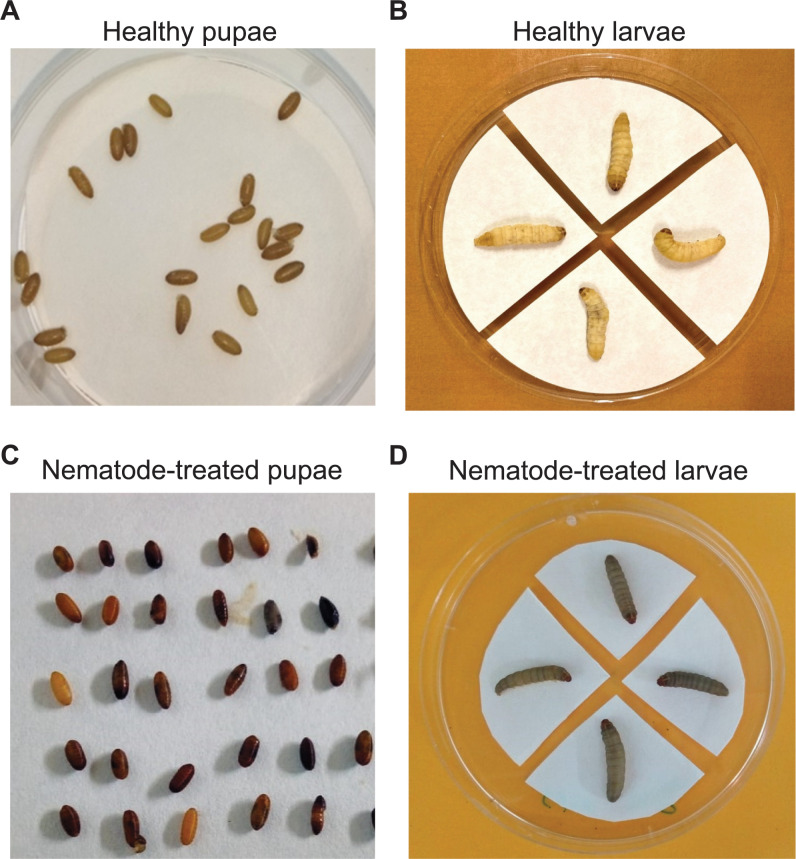
Phenotypic characteristics of healthy insects and of insects exposed to *O*. *tipulae* nematodes. (A) Healthy *C*. *capitata* pupae. (B) Healthy *G*. *mellonella* larvae. (C) *C*. *capitata* pupae exposed to *O*. *tipulae* nematodes. (D) *G*. *mellonella* larvae exposed to *O*. *tipulae* nematodes.

**Fig 3:**
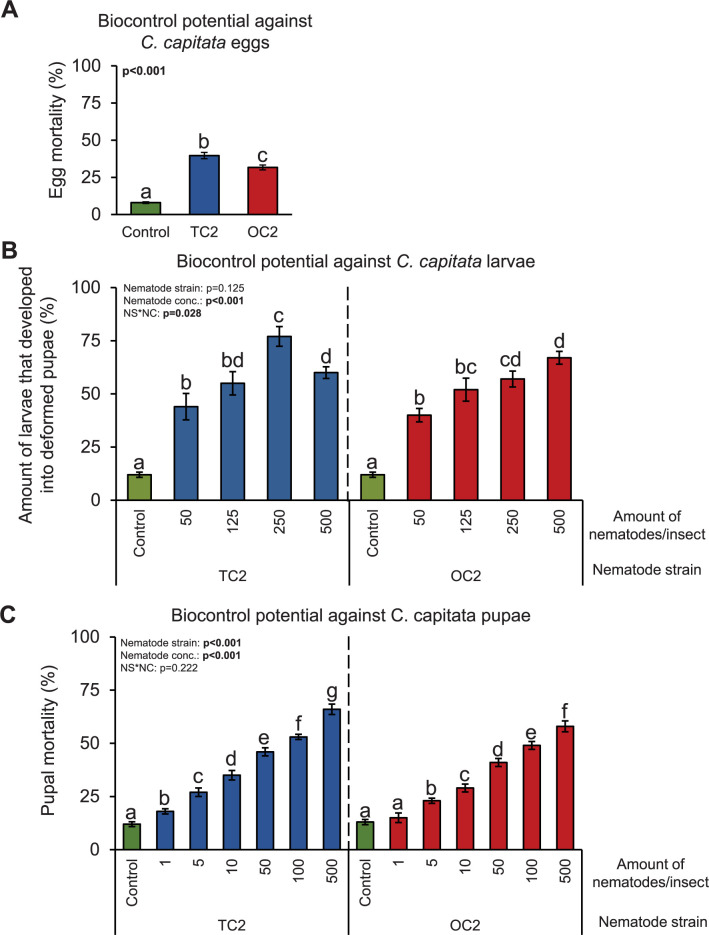
Biocontrol potential of *O*. *tipulae* nematodes against *C*. *capitata* at different developmental stages. (A) Mean (±S.E.M.) mortality of *C*. *capitata* eggs after applying 100 *O*. *tipulae* nematodes/egg. Six Petri plates with 50 eggs each were assayed (n = 6). Different letters indicate significant differences between treatments (P<0.05 by one-way ANOVA with Holm’s multiple comparisons test). (B) Mean (±S.E.M.) per cent of *C*. *capitata* larvae that developed into deformed pupae after applying different amounts of *O*. *tipulae* nematodes. Five Petri plates with 20 larvae each per nematode concentration were assayed (n = 5). Different letters indicate significant differences between treatments within each nematode strain (P<0.05 by two-way ANOVA with Holm’s multiple comparisons test). (C) Mean (±S.E.M.) mortality of *C*. *capitata* pupae after applying different amounts of *O*. *tipulae* nematodes. Five Petri plates with 20 pupae each per nematode concentration were assayed (n = 5). Different letters indicate significant differences between treatments within each nematode strain (P<0.05 by two-way ANOVA with Holm’s multiple comparisons test). All experiments were incubated at 25°C.

**Fig 4:**
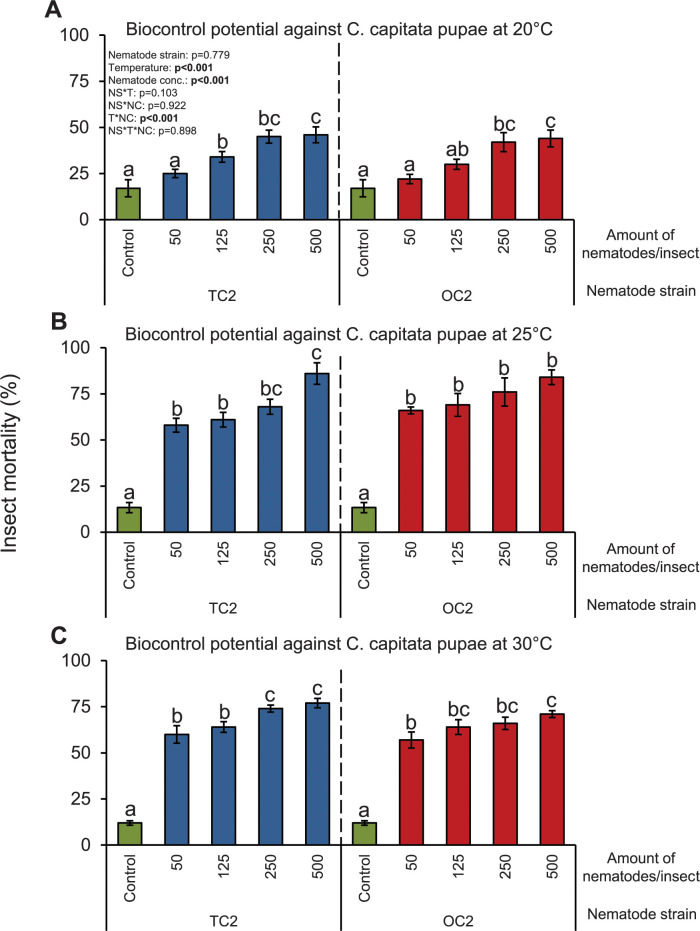
Biocontrol potential of *O*. *tipulae* nematodes against *C*. *capitata* pupae at different temperatures. Mean (±S.E.M.) mortality of *C*. *capitata* pupae after applying different *O*. *tipulae* nematodes at: (A) 20°C, (B) 25°C, and (C) 30°C. Five Petri plates with 20 pupae each per nematode concentration were assayed (n = 5). Different letters indicate significant differences between treatments within each nematode strain and temperature combination (P<0.05 by three-way ANOVA with Holm’s multiple comparisons test).

Insects treated with *O*. *tipulae* TC2 and OC2 show clear symptoms of infestation (Fig 2). The two populations of *O*. *tipulae*, TC2 and OC2, kill or negatively affect the development of *C*. *capitata* eggs, larvae and pupae at all concentrations used (Fig 3). More specifically, TC2 and OC2 nematodes killed 39 and 31% of the eggs, respectively, impaired the metamorphosis of 44 to 77% and of 40 to 67% of the larvae, respectively, and killed between 18 and 66% and between 15 and 58% of the pupae, respectively (Fig 3A–3C). The efficacy of TC2 and OC2 nematodes was particularly high on *C*. *capitata* pupae, and significantly higher mortalities were observed when the pupae were treated with 1 or 5 nematodes/pupa, respectively, compared to the mortalities observed in control treatments (Fig 3C). We therefore conducted additional experiments to confirm our results and evaluated the mortality of *C*. *capitata* pupae exposed to the nematodes at different temperatures. In these additional experiments, we observed that both nematode isolates killed *C*. *capitata* pupae in a concentration- and temperature-dependent manner (Fig 4). Overall, higher mortalities were observed when the pupae were infested with higher number of nematodes and incubated at higher temperatures. The killing potential of both nematode isolates is similar and no significant differences between these two isolates were detected (Fig 4).

### Biocontrol potential of *O*. *tipulae* nematodes against *G*. *mellonella*

**Fig 5:**
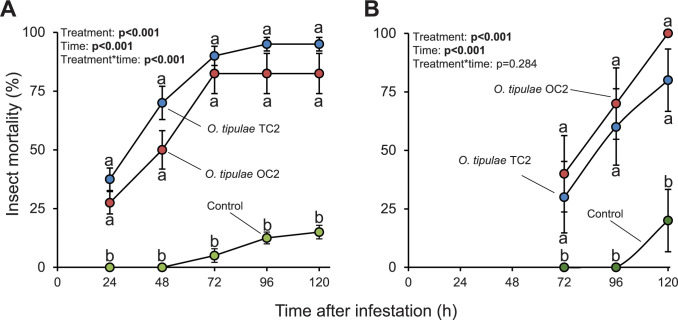
Mortality of *G*. *mellonella* upon infestation by *O*. *tipulae* nematodes. (A) and (B) Mean (±S.E.M.) mortality of *G*. *mellonella* larvae at different time points after applying 500 *O*. *tipulae* nematodes per larva. In experiment 1, four Petri plates with 10 larvae each were assayed (n = 4). In experiment 2, ten Petri plates with 10 larvae each were assayed (n = 10). Different letters indicate significant differences between treatments within time points (P<0.05 by two-way repeated measures ANOVA with Holm’s multiple comparisons test).

**Fig 6:**
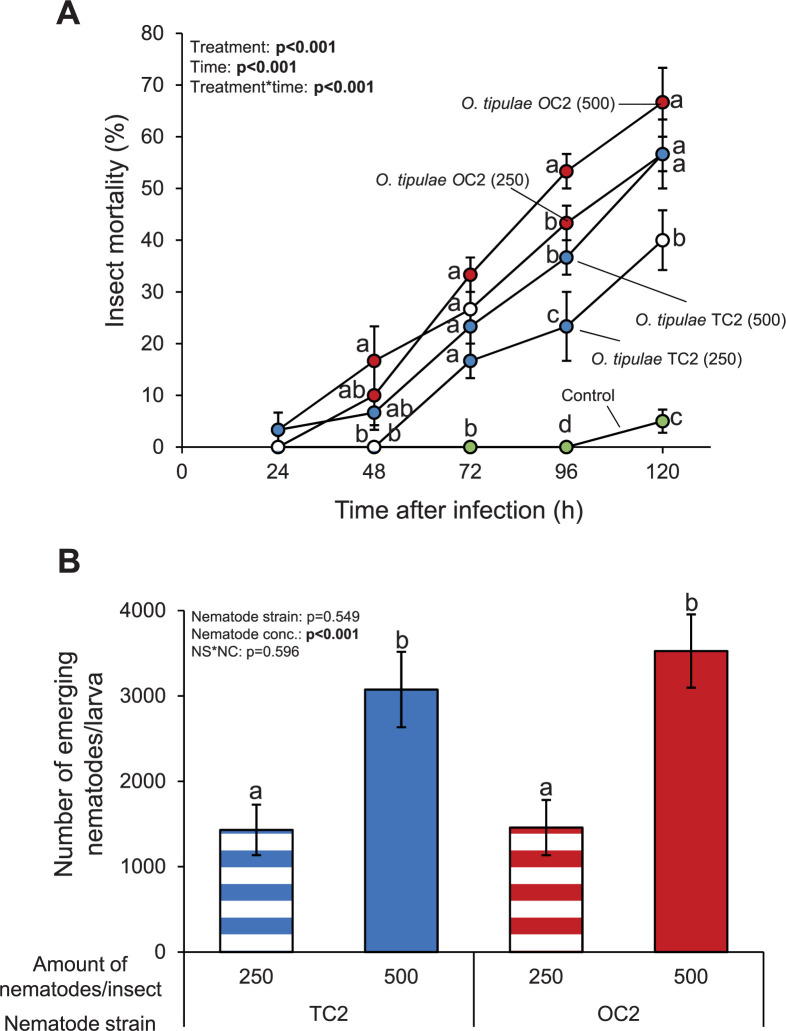
Lethality and reproductive potential of *O*. *tipulae* nematodes in *G*. *mellonella* larvae. (A) Mean (±S.E.M.) mortality of *G*. *mellonella* larvae at different time points after applying either 250 or 500 *O*. *tipulae* nematodes per larva. Three Petri plates with 10 larvae each per nematode concentration were assayed (n = 3). Controls consisted of water treated larvae. Six Petri plates with 10 larvae each were assayed (n = 6). Different letters indicate significant differences between treatments within time points (P<0.05 by two-way repeated measures ANOVA with Holm’s multiple comparisons test). (B) Number of nematodes that emerge from *G*. *mellonella* larvae within ten days after nematode infestation. Infested insects were individually placed in White traps (n = 13–20 per treatment). Different letters indicate significant differences between treatments (P<0.05 by two-way ANOVA with Holm’s multiple comparisons test).

To test the potential of *O*. *tipulae* nematodes to kill other insects apart from *C*. *capitata*, we evaluated the mortality of *G*. *mellonella* exposed to these nematodes. In three independent experiments, both nematode isolates caused high mortality on *G*. *mellonella* larvae, killing between 40 to 100% of the *G*. *mellonella* larvae within 120h post-treatment (Figs 5 and 6). The killing potential of both nematode isolates is similar and only statistically significant differences between these two isolates were detected when the nematodes were applied at 250 nematodes/insect, being OC2 nematodes more lethal than TC2 nematodes (Figs 5 and 6).

### Reproduction potential of *O*. *tipulae* nematodes on *G*. *mellonella*

To evaluate the potential of *O*. *tipulae* nematodes to reproduce on *G*. *mellonella*, we counted the number of emerging nematodes from *G*. *mellonella* dead individuals ten days *post-mortem*. When *G*. *mellonella* larvae were infested with 250 nematodes/insect, around 1400 nematodes/insect emerged from the dead individuals (Fig 6B). When *G*. *mellonella* larvae were infested with 500 nematodes/insect, about 3000–3500 nematodes/insect emerged from the dead insects. No statistically differences were found between the two strains within each concentration.

## Discussion

In this study, several isolates of soil nematodes were recovered from citrus orchards in Tunisia and successfully reared under laboratory conditions. From the recovered nematodes, the potential of two isolates, *O*. *tipulae* TC2 and *O*. *tipulae* OC2, to kill insects was evaluated. Both were able to effectively kill *C*. *capitata* eggs and pupae, to interfere with the metamorphosis of *C*. *capitata* larvae, and to kill and reproduce on *G*. *mellonella* larvae.

Free-living nematodes in the Rhabditida order display different feeding habits such as saprophagous, bacteriophagous, parasitic or even entomopathogenic [53,54]. Some species of *Oscheius* in particular have close associations with insect corpses, either as saprophagous [55] or necromenic nematodes [56], or even kill them [23,27,55]. Similarly, *Acrobeloides nanus* was reported to colonize earthworm cocoons and *Acrobeloides maximus* are frequently recovered from soils baited with insect larvae [57,58]. Moreover, *Caenorhabditis briggsae* and its associated bacteria penetrate, kill and reproduce in insects [59]. Our results are consistent with these earlier reports. *Oscheius*, *Caenorhabditis* and *Acrobeloides* were isolated from Tunisian soils by using an insect baiting technique: one *C*. *elegans* population was isolated from *G*. *mellonella* larvae, one population of *Acrobeloides* sp. was isolated from *G*. *mellonella* larvae and two from *C*. *capitata* pupae, and two populations of *O*. *tipulae* were isolated from *C*. *capitata* pupae, confirming the insect-association nature of these free-living nematodes [23,60].

Nematodes of the family Steinernematidae and Heterorhabditidae are free-living rhabditid nematodes, considered true entomopathogenic nematodes (EPNs), and therefore are used as biocontrol agents against many insect pests [61]. However, other members of the Rhabditida have also shown potential to kill insects and may serve as promising new candidates for biocontrol of insect pests. Several species of *Oscheius* are recognized by their insect-killing abilities and are even often referred to as EPNs, although many of them have not been demonstrated to comply all the conditions that characterize EPNs [62]. Several species have been reported to kill insects and/or to reproduce in them such as: *O*. *basothovii* [31], *O*. *carolinensis* [24], *O*. *chongmingensis* [23,63], *O*. *microvilli* [64], *O*. *myriophilus* [65], *O*. *onirici* [27], *O*. *rugaoensis* [66–68], *O*. *safricana* [69] and *O*. *tipulae* [28]. Some species, although associated to insects, have not been demonstrated to be able to kill them, as it is the case of *O*. *pheropsophi* [34] and *O*. *esperancensis* [70]. Apparently, the insect killing capacity of this genus is more common than expected.

Our results show that two populations of *O*. *tipulae* are capable of killing *C*. *capitata* and *G*. *mellonella*, in a manner that is comparable to highly efficient entomopathogenic nematodes such as *Heterorhabditis* and *Steinernema* [71–73]. Significant mortalities were observed even when one (TC2) or five (OC2) nematodes were used to infest one single *C*. *capitata* pupae. Thus, we add more evidence on the ability of free-living, bacteriophagous nematodes to kill insects, and their potential to complement pest management programs based on these biological control agents [28]. Clearly, more research regarding the feasibility to mass-rear them, and on their performance under field conditions (persistence, abiotic stress tolerance etc.) is required.

Several studies have shown that some free-living nematodes in Rhabditida order interact with EPNs [32,58,74,75]. For instance, *O*. *tipulae*, *O*. *onirici* and *Acrobeloides* spp. have been found to naturally co-occur with EPNs in certain regions of the world and have been isolated in conjunction with EPNs from *G*. *mellonella* cadavers retrieved from baited traps in the soil [32,76,77]. Under which circumstances the use of mixtures of nematode species can be beneficial and increase their efficiency should be carefully evaluated, especially because antagonistic interaction is likely to occur [32,58]. In this study, no species of *Heterorhabditis* and *Steinernema* were found in soil samples, supporting this notion, and suggesting that free-living nematodes might out compete EPNs or that certain edaphic or climatic parameters might favor them over the EPNs.

There is still a lot of controversy on whether or not certain species of free-living nematodes, including *Oscheius* species, can be considered EPNs [62]. An EPN, by definition, is a nematode that has evolved mechanisms to cooperate and associate with insect-pathogenic bacteria to kill insect hosts. It must rapidly kill their hosts with the aid of bacterial partners and must pass on the associated bacteria to future generations [62]. In our study, we clearly show the rapid insect-killing capacity of *O*. *tipulae* nematodes, and also that less than five individuals can cause significant mortality of *C*. *capitata* pupae, but we still do not know if this effect is caused by insect-pathogenic bacteria. The mode of action is therefore uncertain and clearly deserves more attention. Hence, it remains to be determined if *O*. *tipulae* can be considered an EPN, based on Dillman’s definition [62]. Other members of this genus, such as *O*. *chongmingensis* and *O*. *carolinensis* are considered EPNs [23,62]. However, some other authors consider them as facultative scavengers, rather than EPNs, that may be on an evolutionary trajectory leading to an entomopathogenic lifestyle [78]. Similarly, *O*. *tipulae* is considered a facultative kleptoparasite that compete with EPNs for insects [32], an entomophilic nematode [30], and other authors consider this species entomopathogenic [79]. Clearly, more studies are required to resolve this controversy and clarify the ecological classification of these nematodes.

Independently of their ecological classification, we show that *C*. *capitata* and *G*. *mellonella* are highly susceptible to these nematodes. If they rely on closely-associated bacteria to kill remains to be investigated. The entomopathogenic action of some *Oscheius* species has been linked to *Serratia* bacteria which play an essential role during the infection and presumably cause the death of the host insect [80]. For example, *O*. *carolinensis* was consistently associated with four bacterial species, one of which, *Serratia marcescens*, appears to be carried on the cuticle of the nematodes and through its association provides the worms with entomopathogenic potential [25]. Another possibility is that insect’s death is caused simply by tissue damage, or by nematode-produced venom proteins [81,82]. Clearly, the mode of action of these nematodes deserves further attention.

## Conclusion

In conclusion, the present study shows the high control potential of two strains of *O*. *tipulae* against *C*. *capitata*. Under which circumstances and the exact mechanisms how these free-living bacteriophagous nematodes kill insect hosts remain to be investigated. Likewise, their real potential to be used as a biological control agent in the future requires further studies such as extensive field trials.

## Supporting information

S1 Fig Phylogenetic tree based on ribosomal RNA gene sequences of the nematodes isolated in this study and several related species. Phylogenetic relationships based on the nucleotide sequences of the D2-D3 expansion segments of the 28S rRNA gene were inferred by using the Maximum Likelihood method based on the General Time Reversible model. The tree with the highest log likelihood (-6470.16) is shown. The percentage of trees in which the associated taxa clustered together is shown next to the branches. A discrete Gamma distribution was used to model evolutionary rate differences among sites (5 categories (+G, parameter = 0.7981)). The rate variation model allowed for some sites to be evolutionarily invariable ([+I], 19.69% sites). The tree is drawn to scale, with branch lengths measured in the number of substitutions per site. NCBI accession numbers of the sequences used for the analyses are shown.
(PDF)

S2 Fig Phylogenetic tree based on ribosomal RNA gene sequences of the nematodes isolated in this study and several related species. Phylogenetic relationships based on the nucleotide sequences of the internal transcribed spacer (ITS) region of the rRNA gene were inferred by using the Maximum Likelihood method based on the Tamura 3-parameter. The tree with the highest log likelihood (-10573.30) is shown. The percentage of trees in which the associated taxa clustered together is shown next to the branches. A discrete Gamma distribution was used to model evolutionary rate differences among sites (5 categories (+G, parameter = 1.0926)). The rate variation model allowed for some sites to be evolutionarily invariable ([+I], 8.47% sites). The tree is drawn to scale, with branch lengths measured in the number of substitutions per site. NCBI accession numbers of the sequences used for the analyses are shown.
(PDF)

S1 Table NCBI accession numbers of the gene sequences produced in this study. (PDF)

S1 File The data supporting the findings of this study can be found under this link: https://figshare.com/articles/dataset/Data_xlsx/19722223. (XLSX)
