# Supplementary figures and images for: Potential of Oscheius tipulae nematodes as biological control agents against Ceratitis capitata
Source: PLoS One. 2022 Jun 7;17(6):e0269106. doi: 10.1371/journal.pone.0269106 (PMC9200223; doi:10.1371/journal.pone.0269106)

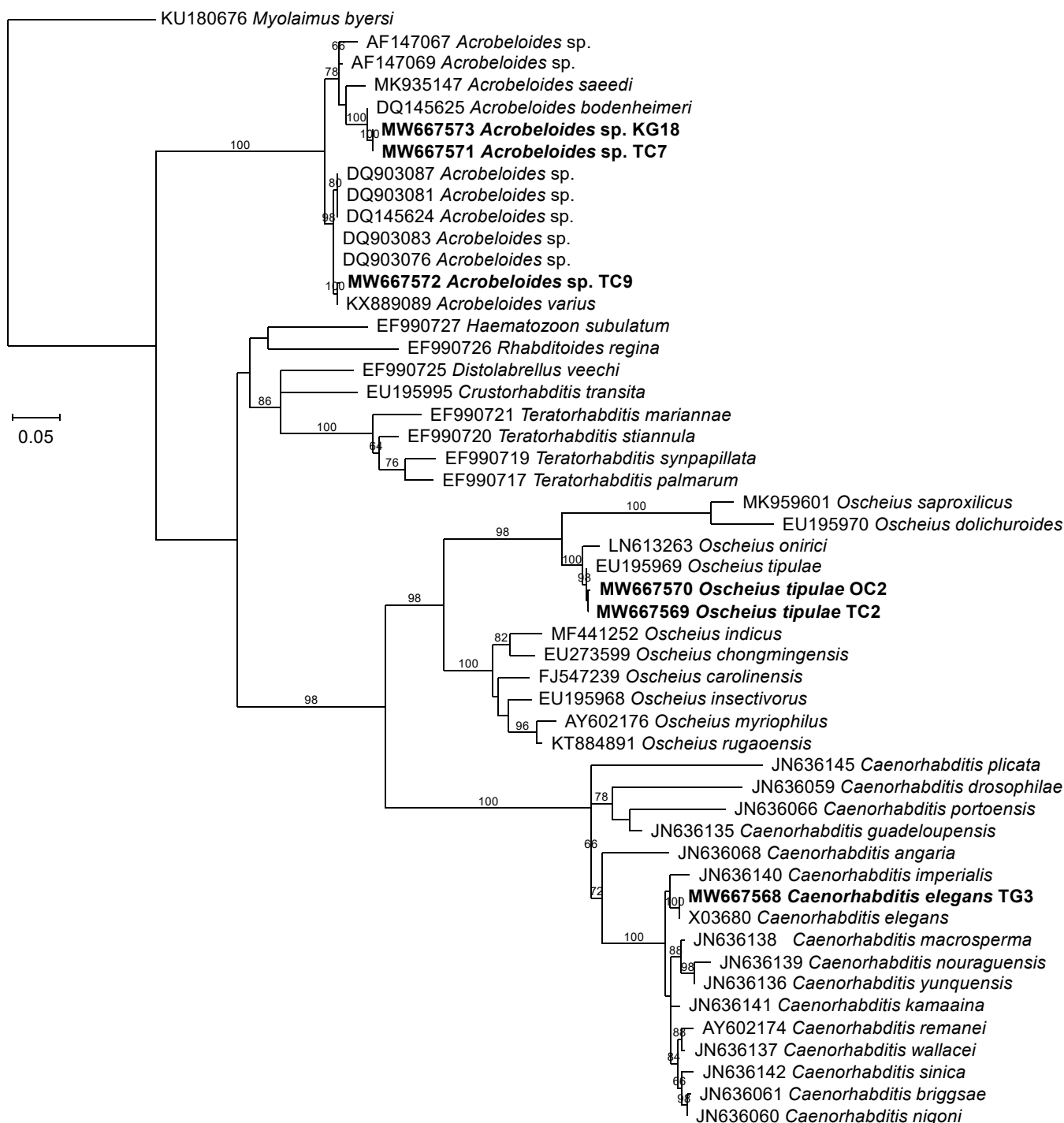

Supplement: S1 Fig — Phylogenetic relationships based on the nucleotide sequences of the D2-D3 expansion segments of the 28S rRNA gene were inferred by using the Maximum Likelihood method based on the General Time Reversible model. The tree with the highest log likelihood (-6470.16) is shown. The percentage of trees in which the associated taxa clustered together is shown next to the branches. A discrete Gamma distribution was used to model evolutionary rate differences among sites (5 categories (+G, parameter = 0.7981)). The rate variation model allowed for some sites to be evolutionarily invariable ([+I], 19.69% sites). The tree is drawn to scale, with branch lengths measured in the number of substitutions per site. NCBI accession numbers of the sequences used for the analyses are shown. (PDF) [file pone.0269106.s001.pdf]

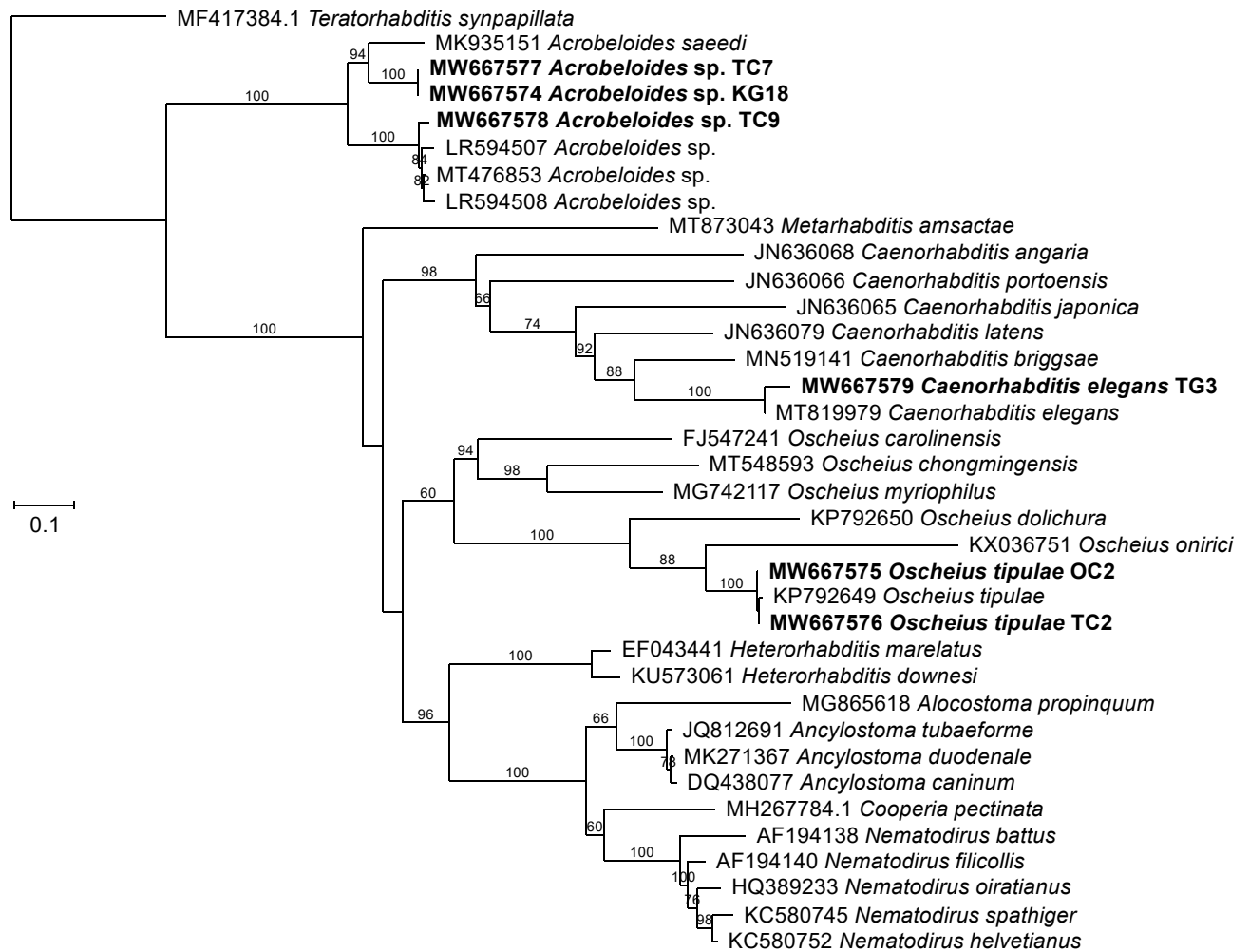

Supplement: S2 Fig — Phylogenetic relationships based on the nucleotide sequences of the internal transcribed spacer (ITS) region of the rRNA gene were inferred by using the Maximum Likelihood method based on the Tamura 3-parameter. The tree with the highest log likelihood (-10573.30) is shown. The percentage of trees in which the associated taxa clustered together is shown next to the branches. A discrete Gamma distribution was used to model evolutionary rate differences among sites (5 categories (+G, parameter = 1.0926)). The rate variation model allowed for some sites to be evolutionarily invariable ([+I], 8.47% sites). The tree is drawn to scale, with branch lengths measured in the number of substitutions per site. NCBI accession numbers of the sequences used for the analyses are shown. (PDF) [file pone.0269106.s002.pdf]
