# Supplementary material for: Potential of Oscheius tipulae nematodes as biological control agents against Ceratitis capitata
Source: PLoS One. 2022 Jun 7;17(6):e0269106. doi: 10.1371/journal.pone.0269106 (PMC9200223; doi:10.1371/journal.pone.0269106)
Supplement: S1 Table — (PDF) [file pone.0269106.s003.pdf]

Supplementary Table 1. NCBI accession numbers of the gene sequences produced in this study.

Organism	rRNA region	NCBI accession number
Caenorhabditis elegans TG3	18S	MW667562
Oscheius tipulae TC2	18S	MW667563
Acrobeloides sp. TC7	18S	MW667564
Acrobeloides sp. TC9	18S	MW667565
Acrobeloides sp. K18g	18S	MW667566
Oscheius tipulae OC2	18S	MW667567
Caenorhabditis elegans TG3	D2D3	MW667568
Oscheius tipulae TC2	D2D3	MW667569
Oscheius tipulae OC2	D2D3	MW667570
Acrobeloides sp. TC7	D2D3	MW667571
Acrobeloides sp. TC9	D2D3	MW667572
Acrobeloides sp. K18g	D2D3	MW667573
Acrobeloides sp. K18g	ITS	MW667574
Oscheius tipulae OC2	ITS	MW667575
Oscheius tipulae TC2	ITS	MW667576
Acrobeloides sp. TC7	ITS	MW667577
Acrobeloides sp. TC9	ITS	MW667578
Caenorhabditis elegans TG3	ITS	MW667579
